# Simulating quantum dynamical phenomena using classical oscillators: Landau-Zener-Stückelberg-Majorana interferometry, latching modulation, and motional averaging

**DOI:** 10.1038/s41598-018-28993-8

**Published:** 2018-08-15

**Authors:** O. V. Ivakhnenko, S. N. Shevchenko, Franco Nori

**Affiliations:** 1 B. Verkin Institute for Low Temperature Physics and Engineering, Kharkov, 61103 Ukraine; 2 V. N. Karazin Kharkov National University, Kharkov, 61022 Ukraine; 3 Theoretical Quantum Physics Laboratory, RIKEN Cluster for Pioneering Research, Wako-shi, Saitama 351-0198 Japan; 4 Physics Department, University of Michigan, Ann Arbor, MI 48109-1040 USA

## Abstract

A quantum system can be driven by either sinusoidal, rectangular, or noisy signals. In the literature, these regimes are referred to as Landau-Zener-Stückelberg-Majorana (LZSM) interferometry, latching modulation, and motional averaging, respectively. We demonstrate that these pronounced and interesting effects are also inherent in the dynamics of classical two-state systems. We discuss how such classical systems are realized using either mechanical, electrical, or optical resonators. In addition to the fundamental interest of such dynamical phenomena linking classical and quantum physics, we believe that these are attractive for the classical analogue simulation of quantum systems.

## Introduction

## Classical-quantum analogies

**Figure 1:**
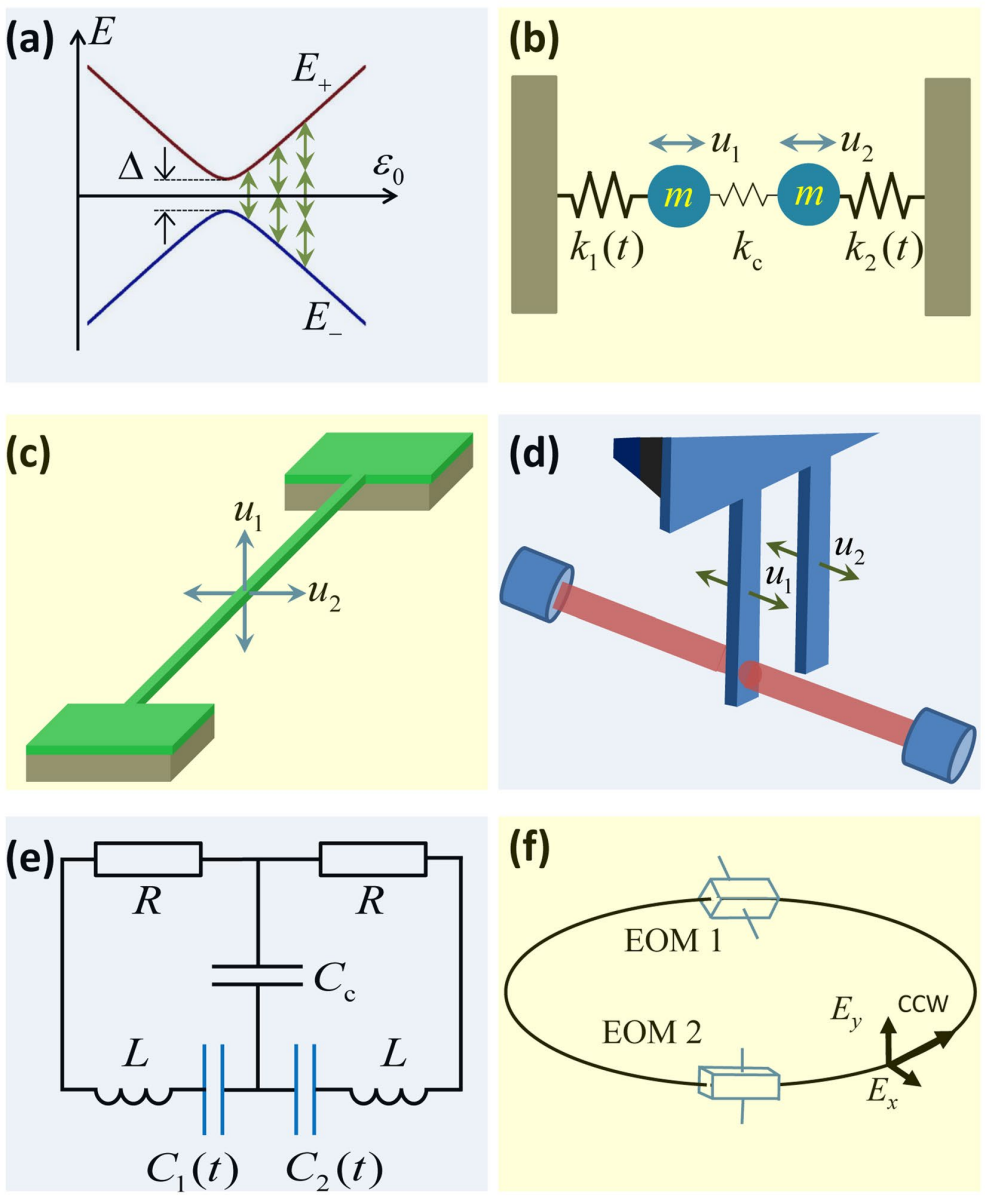
Classical analogues of qubits. In (**a**) the two qubit eigenenergies *E*_±_ are shown to depend on the bias *ε*_0_ and to display avoided-level crossing at *ε*_0_ = 0 with a minimal distance Δ. The system is excited when the characteristic qubit frequency is about a multiple of the driving frequency, Δ*E*/ℏ = *kω*. Several possible classical realizations have been demonstrated: (**b**) two weakly coupled spring oscillators^12^, (**c**) a two-mode nano-beam^14^, (**d**) optomechanical system with two cantilevers^13^, (**e**) two coupled electrical resonators^5,46^, (**f**) two coupled polarization modes in an optical ring resonator^47^.

Classical oscillators are ubiquitous in nature. With some modifications, they provide analogues of systems from other fields of physics. An important example considered here is a basic system of quantum mechanics and quantum technologies: a two-level system, or qubit^1–3^. A qubit is described by its tuned two energy levels, as illustrated in Fig. 1(a). Being driven, such system experiences resonant transitions, which is important for both system characterization and control. However, in a number of works in different contexts, it was argued that diverse classical systems can behave like qubits. Such systems include mechanical, opto-mechanical, electrical, plasmonic, and optical realizations, as illustrated in Fig. 1(b–f).

Models based on classical oscillators were used to describe such phenomena as stimulated resonance Raman effect^4^, electromagnetically induced transparency and Autler-Townes splitting^5,6^, Landau-Zener transitions^7–10^, rapid adiabatic passage^8^, Rabi oscillations^11,12^, Stückelberg interference^13–15^, Fano resonances^16^, squeezed states^17^, strong coupling^18^, light-matter interaction^19^, and dynamical localization^20^. In these works, classical resonators displayed features which are sometimes thought of as fingerprints of quantum coherent behaviour. Also such formulations should be differentiated from the layouts where classical mechanical resonators are used to probe coherent phenomena in quantum systems, like in ref.^21^ (see also ref.^22^), where a classical nanomechanical resonator is used to probe quantum phenomena in superconducting qubits. Nevertheless, we will not discuss other possible realizations of analogues between classical and quantum behaviour, which can also be found for tunneling in Josephson junctions^23–25^ and light propagation in periodic optical structures^26,27^.

The analogy between classical and quantum phenomena is an intriguing and very broad subject, see, e.g., refs^28–33^. However, we would like to limit this work to only classical analogues of strongly-driven qubits. Without taking dissipation into account, a qubit is described by the Schrödinger equation, which (for the pseudospin 1/2) is equivalent to the classical Landau-Lifshitz equation^34^. The extension of this formalism in the presence of dissipation is known as the Landau-Lifshitz-Bloch equation^35–37^. In this way, any classical system which obeys the Landau-Lifshitz equation can mimic a qubit’s behaviour.

Besides being fundamentally interesting, such approach of finding classical-quantum analogies is attractive for the classical analogue simulation of quantum systems (e.g.^38^). In this paper we continue the work in this direction and address other phenomena such as Landau-Zener-Stückelberg-Majorana (LZSM) interferometry^39,40^, latching modulation^41^, and motional averaging^42^.

In the rest of this paper, we first present details of how the description of two classical oscillators can be reduced to the qubit equations of motion. We further consider situations when the mechanical-resonator system is driven by strong periodic or noise signals. We demonstrate the resulting interference fringes, which are remarkably similar to those in the quantum analogues.

## Schrödinger-like classical equation of motion

To be specific, among diverse classical analogues of qubits, we consider mechanical layouts. Such oscillators can be realized as separate resonators^43–45^ or as two modes of one resonator^10,11^. Among the advantages of such mechanical systems are that they operate at room temperature, have small size and high quality factors, and are only weakly coupled to the environment. Such system is described by the equations of motion

1 $$m{\ddot{u}}_{i}+m\gamma {\dot{u}}_{i}+{k}_{i}{u}_{i}+{k}_{{\rm{c}}}({u}_{i}-{u}_{j})=\mathrm{0,}$$

where the displacement *u*_*i*_ relates to the *i*-th oscillator (*i* = 1, 2, *j* ≠ *i*). For oscillators we assume equal effective masses *m*, equal damping rates *γ*, different spring constants ${k}_{i}=m{\omega }_{i}^{2}$, and weak coupling, ${k}_{{\rm{c}}}\ll {k}_{i}$, see Fig. 1(b).

It is important to stress that the other layouts in Fig. 1 are also described by the same equation (1). Therefore, what is written below is equally applicable to any classical two-state system. For example, for two coupled electrical resonators, Fig. 1(e), instead of displacement we have charges on respective capacitances, *u*_*i*_ → *q*_*i*_, the role of masses is played by inductances, *m* → *L*, capacitances replace the spring constants, ${k}_{i}(t)\to {C}_{i}^{-1}(t)$ and *k*_c_ → *C*_c_, and the damping is defined by the resistances, *γ* → *R*/*L*. Thus, the present scheme, inspired by ref.^5^, requires driving via capacitances. Alternatively, the scheme may be modified so that to be driven via inductances, as in ref.^46^. In addition, Fig. 1(d) presents two cantilevers, one of which is coupled to the optical cavity^13^; and in Fig. 1(f) the two polarization modes of the light propagating in counter-clockwise (ccw) direction are shown to be tuned by the electro-optic modulators, EOM1 and EOM2, with the tuning parameter being the electric field inside the EOM1^47^.

To obtain the analogue of a driven qubit, following refs^9,12^, we assume

2 $${k}_{\mathrm{1,2}}={k}_{0}\pm {\rm{\Delta }}k(t\mathrm{)}.$$

Here Δ*k*(*t*) contains, in general, both dc and ac components. Then, introducing the interaction-shifted frequency

3 $${{\rm{\Omega }}}_{0}^{2}=\frac{{k}_{0}+{k}_{{\rm{c}}}}{m},$$

the two Eq. (1) can be rewritten in the matrix form using the notation of the Pauli matrices *σ*_*x*,*z*_:

4 $$(\frac{{d}^{2}}{d{t}^{2}}+\gamma \frac{d}{dt}+{{\rm{\Omega }}}_{0}^{2})\,[\begin{array}{c}{u}_{1}\\ {u}_{2}\end{array}]-(\frac{{k}_{{\rm{c}}}}{m}{\sigma }_{x}+\frac{{\rm{\Delta }}k}{m}{\sigma }_{z})\,[\begin{array}{c}{u}_{1}\\ {u}_{2}\end{array}]=0.$$

Using the ansatz

5 $${\tilde{u}}_{i}={\psi }_{i}\exp (i{{\rm{\Omega }}}_{0}t),\,\,{u}_{i}={\rm{Re}}\,{\tilde{u}}_{i},$$

we obtain the equation

6 $$(\frac{{d}^{2}}{d{t}^{2}}+(\gamma +2i{{\rm{\Omega }}}_{0})\frac{d}{dt}+i{{\rm{\Omega }}}_{0}\gamma )(\begin{array}{c}{\psi }_{1}\\ {\psi }_{2}\end{array})-(\frac{{k}_{{\rm{c}}}}{m}{\sigma }_{x}+\frac{{\rm{\Delta }}k(t)}{m}{\sigma }_{z})(\begin{array}{c}{\psi }_{1}\\ {\psi }_{2}\end{array})=0.$$

Instead of first directly solving these classical equations of motion for specific realizations (as e.g. in refs^5,10,16,46^), we rather demonstrate the equivalence of these to the motion equation of a qubit, and then (in the next section) we will make use of the available solutions. We find this appropriate and pedagogical to first demonstrate the equivalence and then make use of the techniques and solutions available for qubit dynamics. Our approach differs only in details from the other authors’ methods, though. For example, in ref.^12^ the eigenfrequencies are found and only then the Bloch-like equation is obtained, while we do vice versa.

Equation (6) is simplified as follows. First, due to small dissipation, *γ* is neglected next to Ω_0_. Then, the slowly-varying envelope approximation^8,12^ allows neglecting the second derivative (cf. ref.^43^). This means that *ψ*_*n*_ changes little during the time 2*π*/Ω_0_, i.e. its characteristic evolution frequency *ω* is much smaller than Ω_0_, $\omega \ll {{\rm{\Omega }}}_{0}$. Then introducing new notations, from Eq. (6) we obtain

7a $$i\frac{d}{dt}|\psi \rangle =H(t)|\psi \rangle -i\frac{\gamma }{2}|\psi \rangle ,$$

7b $$|\psi \rangle =(\begin{array}{c}{\psi }_{1}\\ {\psi }_{2}\end{array}),$$

7c $$H(t)=\frac{{\rm{\Delta }}}{2}{\sigma }_{x}+\frac{\varepsilon (t)}{2}{\sigma }_{z},$$

7d $${\rm{\Delta }}=\frac{{k}_{{\rm{c}}}}{m{\Omega }_{0}}\,\approx \,\frac{{k}_{{\rm{c}}}}{\sqrt{m{k}_{0}}},$$

7e $$\varepsilon (t)=\frac{{\rm{\Delta }}k(t)}{m{{\rm{\Omega }}}_{0}}\,\approx \,\frac{{\rm{\Delta }}k(t)}{\sqrt{m{k}_{0}}}.$$

In the absence of dissipation, *γ* = 0, equation (7a) formally coincides with the Schrödinger equation for a two-level system with the Hamiltonian *H*(*t*), applying *ħ* = 1.

Dissipation can be eliminated from the problem by the substitution $|\psi \rangle =|\overline{\psi }\rangle \exp (-\frac{\gamma }{2}t)$, then Eq. (7a) becomes

8 $$i\frac{d}{dt}|\overline{\psi }\rangle =H(t)|\overline{\psi }\rangle .$$

Alternatively, the “density matrix” can be introduced as $\rho =|\psi \rangle \langle \psi |$, where $\langle \psi |\,:=\,({\psi }_{1}^{\ast },{\psi }_{2}^{\ast })$. Then for the derivative we obtain

9 $$\dot{\rho }=-\,i[H,\rho ]-\gamma \rho .$$

This coincides with the Bloch equation for a two-level system with the Hamiltonian *H*(*t*), assuming *ħ* = 1, and with equal relaxation rates, *T*_1_ = *T*_2_ = 1/*γ*.

Introducing a convenient parametrization for the “Hamiltonian” and the “density matrix”,

10 $$H(t)=\frac{{\rm{\Delta }}}{2}{\sigma }_{x}+\frac{\varepsilon (t)}{2}{\sigma }_{z}\equiv \frac{1}{2}{\bf{B}}{\boldsymbol{\sigma }},$$

$$\rho =1+X{\sigma }_{x}+Y{\sigma }_{y}+Z{\sigma }_{z}\equiv 1+{\bf{X}}{\boldsymbol{\sigma }},$$

the “Bloch” Eq. (9) can be rewritten in the form of the Landau-Lifshitz-Bloch equation:

11 $$\frac{d}{dt}{\bf{X}}={\bf{B}}\times {\bf{X}}-\gamma {\bf{X}},$$

where

$${\boldsymbol{\sigma }}=({\sigma }_{x},\,{\sigma }_{y},\,{\sigma }_{z}),\,\,{\bf{X}}=(X,\,Y,\,Z),\,\,{\bf{B}}=({\rm{\Delta }},\,0,\,\varepsilon (t)).$$

The diagonal components of the density matrix *ρ* and the *Z*-component of the Bloch vector **X** define the occupation of the respective states, while the off-diagonal components of *ρ* and *X* and *Y* describe the coherence.

We now see the analogy between the classical system and a qubit, described by the Bloch equation. This allows one to expect very similar dynamical phenomena. This was described in the introduction, while specific results are presented in the next two sections. After discussing this analogy, let us point out three key issues (see also, e.g.^11,12^).

First, instead of different energy and phase relaxation rates for a generic quantum two-level system, for a classical analogue they coincide: ${T}_{1}^{-1}={T}_{2}^{-1}=\gamma$. Recall that we have considered identical oscillators, with equal *m*, *k*_0_, and *γ*. In general, all of these quantities should be different. Thus, the equivalence drawn between *T*_1_ and *T*_2_ for the classical system so far is not general. They are only equivalent here because it is assumed that the damping for both oscillators is the same, which in general will not be the case, particularly for different frequency mechanical oscillators. In general, *T*_1_ and *T*_2_ are not related for classical systems, as they would be for a quantum system. For example, 1/*T*_2_ = 1/2*T*_1_ + 1/*T*_*ϕ*_ for a quantum system, but not in the case of two classical coupled oscillators. For two oscillators with only damping rates different, *γ*_1,2_, we would have ${T}_{2}^{-1}=({\gamma }_{1}+{\gamma }_{2}\mathrm{)/2}$.

Second, a qubit at zero temperature relaxes to the ground state, defined by the Bloch vector **X** = (0, 0, 1), while the classical analogue in equilibrium relaxes to the zero Bloch-type vector **X** = (0, 0, 0), as can be seen from Eq. (11). This difference originates from the absence of a classical analogue to the purely quantum process of quantum emission^12^.

Third, one should remember the approximations done: when neglecting the second time derivative we assumed that the classical system’s characteristic evolution frequency *ω* is much smaller than Ω_0_, i. e. $\omega \ll {{\rm{\Omega }}}_{0}$.

We note that the specific parameters depend on the choice of the system, as it was outlined in the introduction. To show specific numbers, we can take the ones close to ref.^10^ for a nanomechanical two-mode beam: $m\simeq {10}^{-15}$ g, ${k}_{0}\simeq \mathrm{3\ }$ N/m, ${k}_{{\rm{c}}}\simeq \mathrm{0.003\ }$ N/m $\ll {k}_{0}$, $\gamma \simeq 80$ Hz ⋅ 2*π*. These parameters give the following: ${{\rm{\Omega }}}_{0}\simeq 6$ MHz ⋅ 2*π*, which indeed satisfies ${{\rm{\Omega }}}_{0}\gg \gamma$, then, ${\rm{\Delta }}\simeq 7$ kHz ⋅ 2*π*, which makes driving with $\omega \sim {\rm{\Delta }}$ feasible, and also ${\rm{\Delta }}k\sim \mathrm{0.03\ }$ N/m, which allows to discuss the regime of strong driving, with the amplitude $A\gg \omega ,{\rm{\Delta }}$.

## Solutions of the Schrödinger-like equation

Using the analogy between the dynamical equations for the two coupled mechanical resonators and a two-level system, one can rewrite results from the respective publications, e.g. from refs^39,48,49^. For convenience, some analytical results are written down below, while detailed solutions are illustrated in the next Section.

For the stationary Hamiltonian (7c) with the time-independent bias *ε* = *ε*_0_, we can transform from the functions *ψ*_*i*_ to *ψ*_±_, which define the eigenstates, analogously to the diagonalization of the Hamiltonian for qubits, e.g. ref.^39^:

12 $$i\frac{d}{dt}(\begin{array}{c}{\psi }_{-}\\ {\psi }_{+}\end{array})=-\,\frac{{\omega }_{0}}{2}{\sigma }_{z}(\begin{array}{c}{\psi }_{-}\\ {\psi }_{+}\end{array}),$$

$${\omega }_{0}=\sqrt{{{\rm{\Delta }}}^{2}+{\varepsilon }_{0}^{2}}.$$

The solution of these two uncoupled equations is

$${\psi }_{\pm }(t)={\psi }_{\pm }\mathrm{(0)}\exp (\mp i\frac{{\omega }_{0}}{2}t).$$

Together with the ansatz (5), this gives eigenfrequencies for the oscillations:

13 $${{\rm{\Omega }}}_{\pm }={{\rm{\Omega }}}_{0}\mp \frac{{\omega }_{0}}{2}={{\rm{\Omega }}}_{0}\mp \frac{1}{2}\sqrt{{{\rm{\Delta }}}^{2}+{\varepsilon }_{0}^{2}},$$

which display the avoided-level crossing at the zero offset *ε*_0_ = 0. (This coincides with the eigenfrequencies from ref.^12^, assuming ${\rm{\Delta }}\omega \ll$ Ω_0_, i.e. ${{\rm{\Omega }}}_{d},{{\rm{\Omega }}}_{c}\ll {{\rm{\Omega }}}_{0}$ therein.)

The eigen-functions *ψ*_±_ are the amplitudes of the respective eigen-modes. These are analogous to the energy-level occupation amplitudes for qubits. Accordingly, we will be interested in the “occupation” of one of the modes, namely, |*ψ*_+_|^2^, which can be related to a qubit upper-level occupation probability. In experiments such value can be probed as an amplitude of the oscillations of the respective mode^10^. For example, if initially one, say “−”, mode is excited, the problem can be formulated in finding the amplitude of the other mode; in this sense, the value |*ψ*_+_|^2^ can be interpreted as a transition probability, describing the transition from one mode to another.

Consider now several regimes for the dc + ac driving:

14 $$\varepsilon (t)={\varepsilon }_{0}+A\,\sin \,\omega t.$$

For the single passage of the avoided crossing, we have the Landau-Zener problem, for which the solution is given by the probability

15 $${|{\psi }_{+}|}^{2}={P}_{{\rm{LZ}}}=\exp (\,-\,2\pi \delta ),$$

$$\delta =\frac{{{\rm{\Delta }}}^{2}}{4v},$$

$$v=A\omega \sqrt{1-{({\varepsilon }_{0}/A)}^{2}}.$$

This coincides with the result for the LZ-problem in refs^9,10^ with the linear driving: *v* = *α* = *Aω*.

Analogously, for the double-passage problem, we have the Stückelberg oscillations:

16 $${|{\psi }_{+}|}^{2}=4{P}_{{\rm{LZ}}}(1-{P}_{{\rm{LZ}}}){\mathrm{Sin}}^{2}{{\rm{\Phi }}}_{{\rm{St}}},$$

$${{\rm{\Phi }}}_{{\rm{St}}}=\frac{1}{2}{\int }_{{t}_{1}}^{{t}_{2}}\sqrt{{{\rm{\Delta }}}^{2}+\varepsilon {(t)}^{2}}\,dt+{\tilde{{\phi }}}_{{\rm{S}}},$$

where ${\tilde{{\phi }}}_{{\rm{S}}}={\tilde{{\phi }}}_{{\rm{S}}}(\delta )$ is a parameter varying from −*π*/4, for $\delta \ll 1$, to −*π*/2, for $\delta \gg 1$^15^. We note that the integral above can be estimated, first, by neglecting Δ, and, second, for *ε*_0_ = 0, then the integral is given by a special function: a full elliptic integral of the second kind^50^.

For the multiple-passage problem, consider here only the fast-passage (small Δ) limit. Then multi-photon Rabi oscillations are envisaged; next to the *k*-th resonance, where ${\varepsilon }_{0}\sim k\omega$, we have

17 $${|{\psi }_{+}^{(k)}(t)|}^{2}=\frac{1}{2}\frac{{{\rm{\Delta }}}_{k}^{2}}{{{\rm{\Omega }}}_{{\rm{R}}}^{(k\mathrm{)2}}}(1-\,\cos \,{{\rm{\Omega }}}_{{\rm{R}}}^{(k)}t),$$

$${{\rm{\Delta }}}_{k}={\rm{\Delta }}{J}_{k}(A/\omega ),$$

$${{\rm{\Omega }}}_{{\rm{R}}}^{(k)}=\sqrt{{{\rm{\Delta }}}_{k}^{2}+{(k\omega -|{\varepsilon }_{0}|)}^{2}}.$$

For large arguments, the Bessel function has the oscillating asymptote ${J}_{k}(x)\approx \sqrt{\mathrm{2/}\pi x}\,{\rm{co}}s[x-(2k+1)\pi \mathrm{/4}]$. If necessary, the damping is described by adding the factor exp(−*γt*) in Eqs (16,17). The time-averaged probability distribution is given by the series of Lorentzians:

18 $$\overline{{|{\psi }_{+}|}^{2}}=\sum _{k}\frac{{{\rm{\Delta }}}_{k}^{2}}{{{\rm{\Delta }}}_{k}^{2}+{(k\omega -|{\varepsilon }_{0}|)}^{2}+{\gamma }^{2}}.$$

This, when plotted as a function of *ε*_0_ and *A*, could serve as a visualization of the LZSM interference, which is the main subject of the next Section.

An analysis of the above equations allows for interpretations of specific systems. In our example of two coupled classical oscillators, we have for the Rabi frequency:

19 $${{\rm{\Omega }}}_{{\rm{R}}}={{\rm{\Omega }}}_{{\rm{R}}}^{\mathrm{(1)}}\sim {\rm{\Delta }}\propto {k}_{{\rm{c}}},$$

which means that the energy transfer between the two oscillators, or between the two modes of a mechanical resonator, appears with a rate proportional to the coupling strength^4^.

## Dynamics and interference in the classical two-state system

The Schrödinger-type and Bloch-type equations were presented above for two coupled mechanical resonators. This was done with several assumptions, which allowed reducing the original Eq. (4) to Eq. (7a). From the latter, a qubit-like behaviour follows. In this section we confirm and demonstrate this, by solving numerically the original equations (4). We consider a driven system with the bias *ε*(*t*) = *ε*_0_ + *ε*_1_(*t*), where *ε*_1_(*t*) is one of the following:

1. the sinusoidal function *ε*_1_(*t*) = *A* sin *ωt* with weak or strong amplitudes, so-called Rabi and LZSM regimes, respectively;
2. the rectangular driving with *ε*_1_(*t*) = *A* sgn(sin *ωt*), so-called latching modulation; and
3. a noisy signal, corresponding to random jumps between *ε*_1_ = +*A* and −*A*, with the characteristic switching frequency *χ*.

### Rabi oscillations

**Figure 2:**
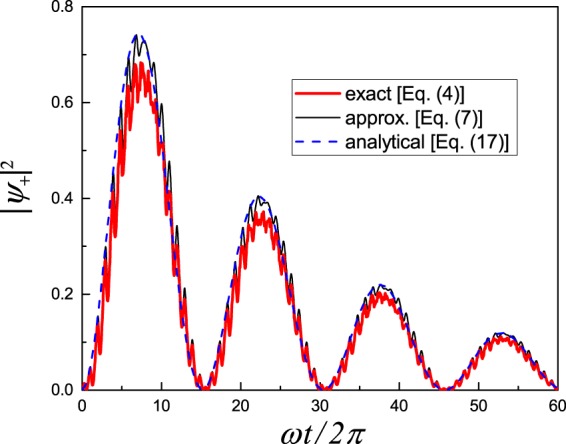
Classical Rabi-like oscillations. When a two-state system is driven by a resonant signal, with *ω* = *ω*_0_, the mode occupation |*ψ*_+_|^2^ displays damping oscillations with Rabi frequency Ω_R_. The three curves display the solutions of the exact equations (4), the approximate ones (7), and the analytical solution, Eq. (17).

Let us first consider the Rabi regime with a weak-amplitude sinusoidal driving. For calculations, we here choose the resonant frequency, *ω* = *ω*_0_ with *ε*_0_ = 5Δ, weak amplitude *A* = 0.7*ω*, and significant relaxation *γ* = 0.006*ω*, in order to see the damping of the Rabi oscillations. In Fig. 2, the thick red curve shows the numerical solution of the exact Eq. (6), the thin black curve is for the numerical solution of the approximate Schrödinger-like Eq. (7a), and the dashed blue curve depicts the analytical solution, Eq. (17). Similarly to their quantum counterparts, classical oscillations appear at the same conditions, of weak resonant driving, and have a similar expression for the Rabi frequency, Eq. (17). An important distinction is that the oscillations relax to zero, in contrast to the quantum case, where resonant oscillations result in a steady state with nonzero population of the excited state. As we can conclude from Fig. 2, for non-trivial results we should average before the system relaxes. This means that the analogue simulation of the quantum system has to be realized as the dynamics of the classical system on time scales Δ*t* < *γ*^−1^. In particular, for the resonant excitation, like the one in Fig. 2, after averaging the oscillating dynamics, we obtain $\overline{{|{\psi }_{+}|}^{2}}\sim 0.5$ for $\gamma {\rm{\Delta }}t\ll 1$, while this occupation decreases for increasing *γ*Δ*t*.

### LZSM interferometry

This regime occurs when a two-level system is strongly driven by a sinusoidal signal and monitored when changing its parameters; see ref.^39^ and references therein. In such processes one is interested in the excitation probability, which increases periodically due to LZSM transitions between the states. After time averaging, the excitation probability displays increased values in the vicinity of the multi-photon resonances, where the energy difference between the states is matched by multiples of the driving frequency. Changing the system parameters, one can plot such interference fringes. This approach is useful both for controlling the system state and for defining the system parameters and its coupling to the environment^49,51^.

**Figure 3:**
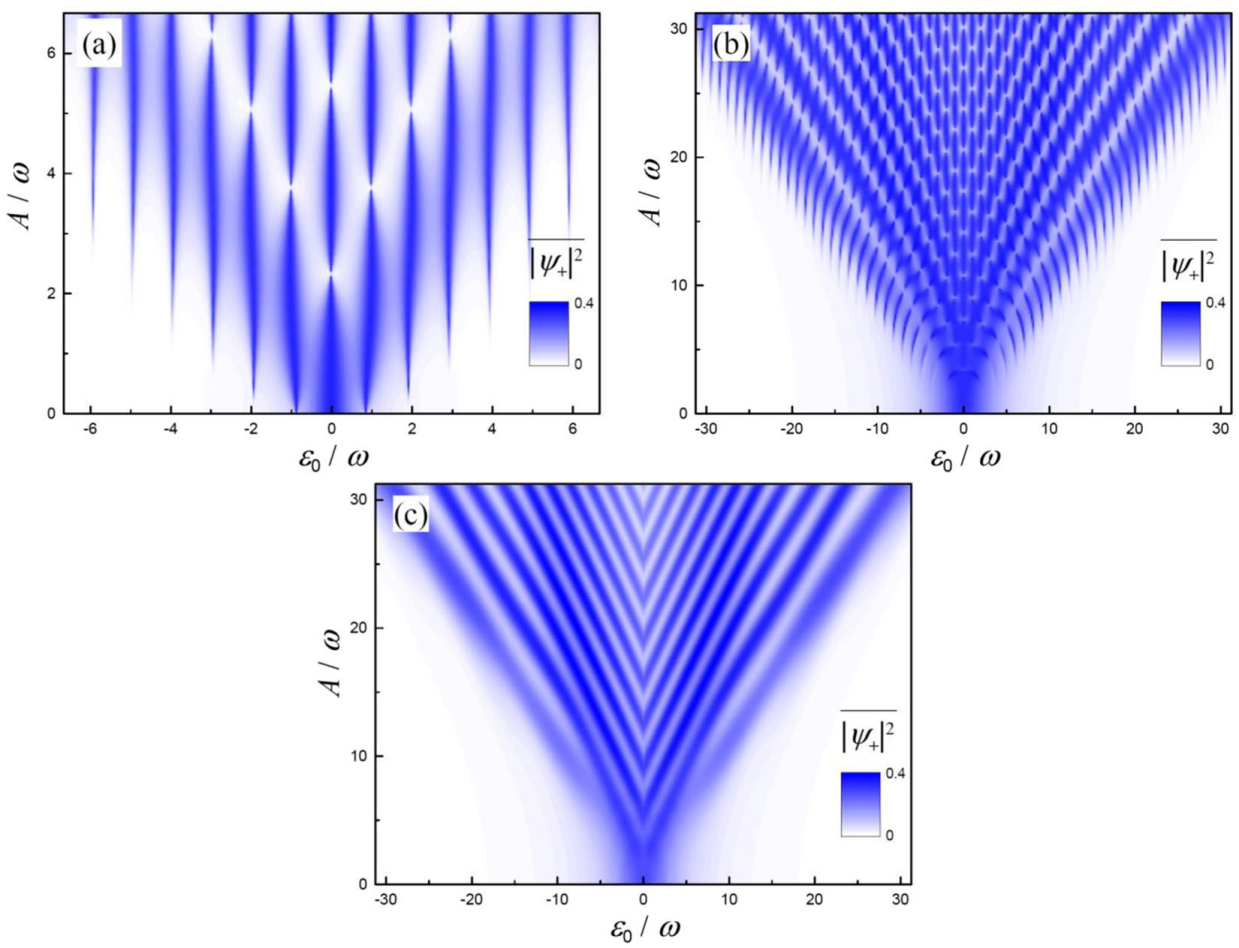
LZSM interferogram for two *classical* oscillators. In (**a**) and (**b**) we present the fast- and slow-driving cases with *ω*/Δ = 2 and 1/3, respectively, for *small* damping, while panel (c) demonstrates the case of *stronger* damping for *ω*/Δ = 1/3; see main text for details. Note that analogous LZSM interferograms were studied for qubits in ref.^39^.

To demonstrate this regime, we now consider the classical-oscillators system in the strong-driving regime, where, for a driven qubit in the same regime, LZSM interference takes place. Figure 3 shows the time-averaged mode occupation $\overline{{|{\psi }_{+}|}^{2}}$ as a function of the bias *ε*_0_ and the driving amplitude *A*. With the numerical solution of the exact Eq. (6) for relatively high and low frequencies $(\omega \,\gtrless \,{\rm{\Delta }})$, we obtain the interferograms in Fig. 3(a,b), respectively. We can observe an important feature in these graphs: they display that the excitations appear at the position |*ε*_0_| = *kω*, with an integer *k*, analogously to the multi-photon transitions for qubits, as illustrated by the arrows in Fig. 1(a). These correspond to the minima of the denominator in Eq. (18), while the narrowing of these resonance lines appears around the zeros of the Bessel functions, entering in the numerators in Eq. (18). Note that this results in the interruptions of the resonance lines, where there are no transitions, even though the resonance condition, |*ε*_0_| = *kω*, is fulfilled. This phenomena for a quantum system is known as the coherent destruction of tunneling^52–54^.

Note that the interferograms in Fig. 3(a,b) are analogous to the ones obtained for diverse qubit systems, see ref.^39^ and references therein. In particular, the interferograms in Fig. 3(a,b) are analogous to Fig. 7(b) and Fig. 8(b) in ref.^39^, respectively. See also ref.^55^ where an analogous interferogram was recently calculated for a quantum-dot-electromechanical device.

Furthermore, Fig. 3(c) demonstrates the case of relatively stronger damping, when the interference fringes appear mostly due to the neighboring two transitions of the avoided crossing in Fig. 1(a). In this case, the resonances in Fig. 3(c) form characteristic V-shaped lines. We can observe that the outer lines are inclined along *A* = ±*ε*_0_, so that there is no excitation beyond these lines. This is because at small driving amplitudes, *A* < |*ε*_0_|, the avoided crossing in Fig. 1(a) is not reached, which explains the absence of the excitation. This regime of relatively strong damping with the V-shaped fringes was called quasiclassical in ref.^56^, which was studied for superconducting and semiconducting qubits in refs^40,56^.

In Fig. 3 we have chosen the following parameters for calculations: relatively weak damping in (a) and (b), *γ* = 0.02⋅*ω*/2*π* and *γ* = 0.1 ⋅ *ω*/2*π*, respectively, and stronger damping in (c), *γ* = *ω*/2*π*. The initial occupation was zero, *ψ*_+_(*t* = 0) = 0, like in Fig. 2, and then we averaged for the time interval ${\rm{\Delta }}t\sim {\gamma }^{-1}$. Namely, we took Δ*t* = 50, 8, 1 ⋅ 2*π*/*ω* for the three panels in Fig. 3, respectively. If we choose $\gamma {\rm{\Delta }}t\ll 1$ or $\gamma {\rm{\Delta }}t\gg 1$, we would obtain similar data as in Fig. 3, but with a maximum amplitude closer to 0.5 or 0, respectively. After discussing this here, for the rest of the interferograms below we will assume $\gamma {\rm{\Delta }}t\ll 1$.

**Figure 4:**
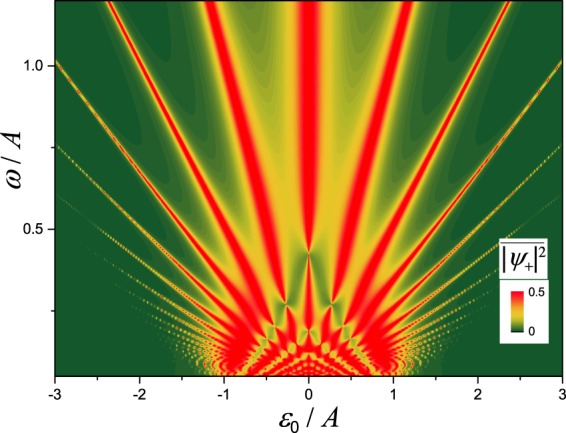
LZSM interferogram showing the dependence of the time-averaged mode occupation $\overline{{|{\psi }_{+}|}^{2}}$ on the driving frequency *ω* and bias *ε*_0_. Note that the analogous interferogram for a qubit was experimentally observed in ref.^41^.

Alternatively, in addition to the above interferograms, one may be interested in the dependence on the driving frequency *ω*, as in refs^41,42^. We present such diagram in Fig. 4. Here the driving amplitude *A* is considered constant and it is used for normalization, which differs from the interferogram in the previous figure, where the driving amplitude was the variable value.

### Latching modulation

If the system is driven by rectangular pulses instead of a sinusoidal signal, it experiences periodic fast changes between the two states. Namely, if the bias is *ε*(*t*) = *ε*_0_ + *A*sgn(sin *ωt*), this means that the system is abruptly latched between the two limiting states with the bias given by either *ε*_0_ + *A* or *ε*_0_ − *A*, where it stays in each for about half of the period^41^. A conceptual distinction from the sinusoidal driving is in that the avoided-level crossing, where LZSM transitions appear, is crossed rapidly. That is why the theory developed for smooth sinusoidal driving had to be revisited for the rectangular-pulse driving, which was done in ref.^41^ both theoretically and experimentally for a superconducting qubit. Here, we study such a formulation for our classical system: two coupled classical oscillators.

**Figure 5:**
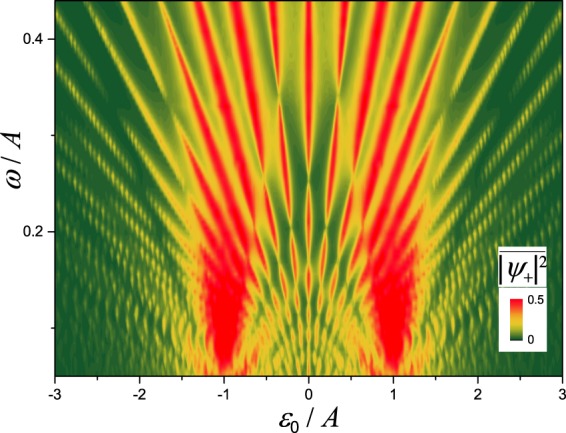
Latching modulation with two classical oscillators. The time-averaged mode occupation $\overline{{|{\psi }_{+}|}^{2}}$ is plotted when the system is driven by rectangular pulses. Note that the analogous interferogram for a qubit was experimentally observed in ref.^42^.

To describe this regime, we have solved Eq. (6), as in the previous subsection, but now with a different bias. Figure 5 shows the time-averaged mode occupation $\overline{{|{\psi }_{+}|}^{2}}$ as a function of the bias *ε*_0_ and the driving frequency *ω*. Such latching modulation displays interference fringes, different from the ones shown above, for a harmonic driving. Interestingly, at low frequencies the system is indeed mostly latched to the two states with the resonances around *ε*_0_ = ±*A*, while for higher frequencies there is no trace of the latching, and the position of the resonances is described by the inclined resonance lines. This means that, due to the interference, our system latching between *ε*_0_ = ±*A* displays resonances for any other values of *ε*_0_. Note that here, for the classical system, we obtained a remarkable agreement with the diagram obtained recently for the experimental qubit in ref.^41^.

### Motional averaging

Differently from above, in the regime which we refer to as the motional averaging, the system is driven by noise rather than by a periodic signal. The rapid jumps between the two states appear stochastically. For this we follow ref.^42^, where it was demonstrated for a qubit that when increasing the frequency of the jumps, two separate spectral lines merge into one line. This observation allowed the authors of ref.^42^ to speculate about the analogue simulation of this so-called motional averaging, originally observed in nuclear magnetic resonance spectroscopy.

**Figure 6:**
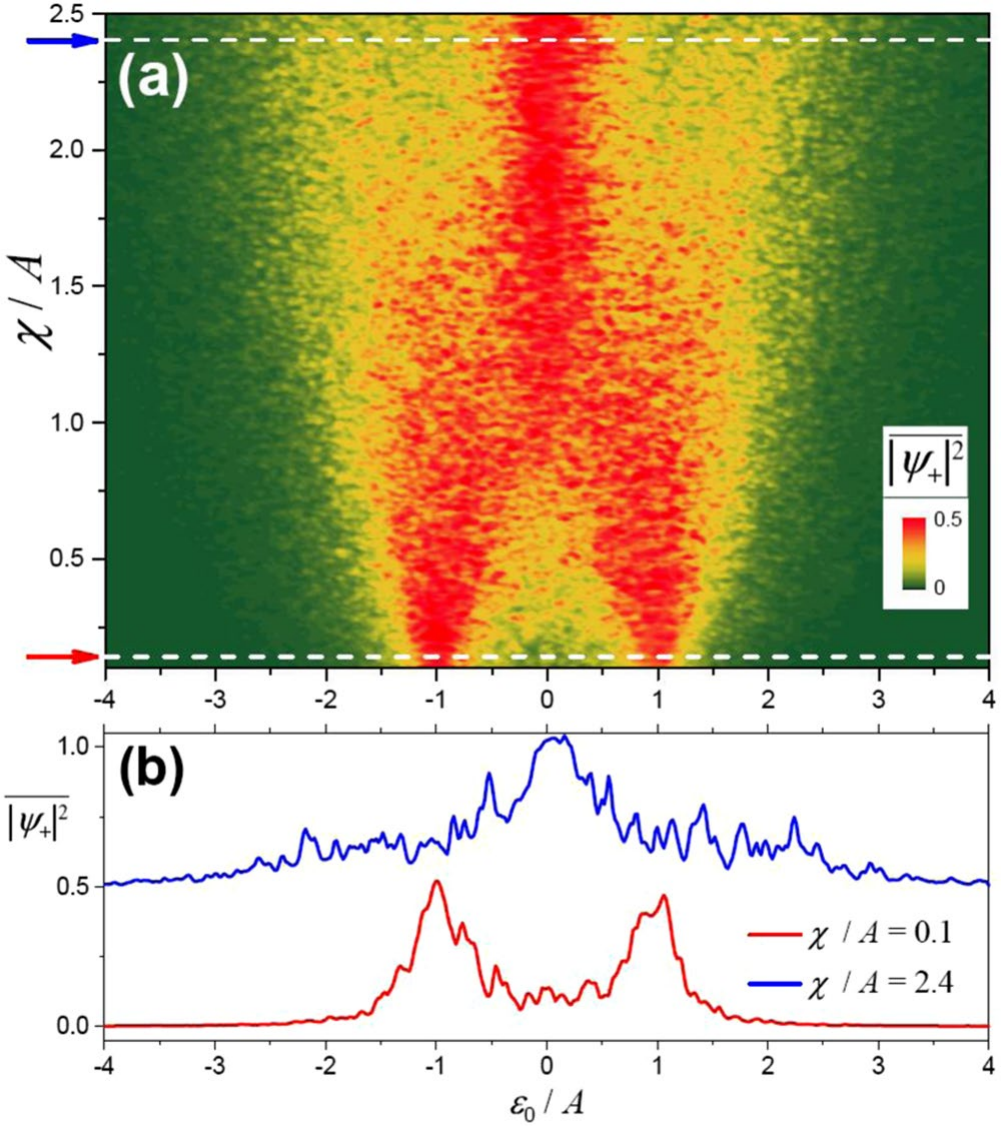
Motional averaging with two classical oscillators. (**a**) The time-averaged mode occupation $\overline{{|{\psi }_{+}|}^{2}}$ as a function of the bias *ε*_0_ and the jumping rate *χ*; this displays two peaks at around *ε*_0_ = ±*A* for a slowly jumping signal with $\chi \ll A$, while the faster jumping, with $\chi \gtrsim A$, results in the merging of the two peaks into one. (**b**) The time-averaged mode occupation as a function of the bias *ε*_0_ for the two values of the jumping rate *χ*. Note that analogous dependencies were experimentally demonstrated for a superconducting qubit in ref.^42^.

Accordingly, we consider here the classical two-state system driven by a non-periodic signal. Namely, we take the bias *ε*(*t*) = *ε*_0_ + *ε*_1_(*t*), admitting random jumps between *ε*_1_ = +*A* and −*A*. The jumps are assumed to appear with the average jumping rate *χ*. In Fig. 6 we plot the time-averaged mode occupation $\overline{{|{\psi }_{+}|}^{2}}$ as a function of the detuning *ε*_0_ and the characteristic switching frequency *χ*.

Our data shown in Fig. 6 are consistent with the result of ref.^42^ in that, at low *χ*, there are two peaks, which merge into one, for increasing *χ*. To make this point explicit, in Fig. 6(b) we plot the two cross-sections of Fig. 6(a) along the horizontal white dashed lines. The red curve in Fig. 6(b) is plotted for a low switching frequency, and this displays two distinct peaks at *ε*_0_ = ±*A*. The blue curve, shifted vertically for clarity, is plotted for a relatively high switching frequency. This displays a main peak aroung *ε*_0_ = 0, which can be interpreted as the averaging due to the motion between the two states.

## Conclusions

Remarkably, the classical system of two weakly coupled classical oscillators form the doppelgänger of a quantum two-level system. Namely, its equation of motion formally coincides with either the Schrödinger equation or with the Bloch equation in the cases when the relaxation is either ignored or taken into account, respectively. This means that the dynamical phenomena of the two-state classical system can be directly described by the ones already studied for the quantum two-level systems, and vice versa. This was known and studied some time ago, e.g. in refs^4,5,7,47^. However, diverse experimental mechanical resonators which are good enough for analogue simulations appeared only recently^11,13,43,44^. Such mechanical resonators have high quality factors and have reliable control of their inter-mode coupling. Moreover, there is recent interest in strongly-driven quantum systems, e.g. refs^39,41,42^. These two developments stimulated us to further consider the analogy between weakly and controllably coupled mechanical oscillators and the driven quantum few-level system. In particular, we demonstrated classical analogues of the effects recently studied for qubits: Landau-Zener-Stückelberg-Majorana interferometry, latching modulation, and motional averaging. Besides the pure interest of such dynamical phenomena linking classical and quantum physics, one may consider simulating some quantum phenomena with classical systems.
